# Quantitative Mass Spectrometry Analysis Using PAcIFIC for the Identification of Plasma Diagnostic Biomarkers for Abdominal Aortic Aneurysm

**DOI:** 10.1371/journal.pone.0028698

**Published:** 2011-12-07

**Authors:** Adelina E. Acosta-Martin, Alexandre Panchaud, Maggy Chwastyniak, Annabelle Dupont, Francis Juthier, Corinne Gautier, Brigitte Jude, Philippe Amouyel, David R. Goodlett, Florence Pinet

**Affiliations:** 1 INSERM, U744, Lille, France; 2 University of Lille Nord de France, USDL, Lille, France; 3 Institut Pasteur de Lille, Lille, France; 4 Medicinal Chemistry, University of Washington, Seattle, Washington, United States of America; 5 Centre Hospitalier Regional et Universitaire, Lille, France; I2MC INSERM UMR U1048, France

## Abstract

**Background:**

Abdominal aortic aneurysm (AAA) is characterized by increased aortic vessel wall diameter (>1.5 times normal) and loss of parallelism. This disease is responsible for 1–4% mortality occurring on rupture in males older than 65 years. Due to its asymptomatic nature, proteomic techniques were used to search for diagnostic biomarkers that might allow surgical intervention under nonlife threatening conditions.

**Methodology/Principal Findings:**

Pooled human plasma samples of 17 AAA and 17 control patients were depleted of the most abundant proteins and compared using a data-independent shotgun proteomic strategy, Precursor Acquisition Independent From Ion Count (PAcIFIC), combined with spectral counting and isobaric tandem mass tags. Both quantitative methods collectively identified 80 proteins as statistically differentially abundant between AAA and control patients. Among differentially abundant proteins, a subgroup of 19 was selected according to Gene Ontology classification and implication in AAA for verification by Western blot (WB) in the same 34 individual plasma samples that comprised the pools. From the 19 proteins, 12 were detected by WB. Five of them were verified to be differentially up-regulated in individual plasma of AAA patients: adiponectin, extracellular superoxide dismutase, protein AMBP, kallistatin and carboxypeptidase B2.

**Conclusions/Significance:**

Plasma depletion of high abundance proteins combined with quantitative PAcIFIC analysis offered an efficient and sensitive tool for the screening of new potential biomarkers of AAA. However, WB analysis to verify the 19 PAcIFIC identified proteins of interest proved inconclusive save for five proteins. We discuss these five in terms of their potential relevance as biological markers for use in AAA screening of population at risk.

## Introduction

Abdominal aortic aneurysm (AAA) is one of the leading causes of death in industrialized countries where a growing percentage of the population is over 65 years of age [1]. AAA is a vascular pathology characterized by an increase of aorta diameter to at least 1.5 times the diameter of a standard reference or ≥30 mm of infrarenal aorta diameter, and a loss of parallelism of the aortic wall at the infrarenal region. Known risk factors for AAA are advanced age, male gender, cigarette smoking, hypertension, genetic susceptibility and the presence of another atherosclerotic localization [2]. Hence AAA primarily affects elderly males at a prevalence of 5% with ruptures being responsible for 1–4% of the total mortality in males older than 65 years. Moreover, mortality occurs in 65–75% of patients before they arrive at hospital [3]. This high mortality is largely due to the fact that the vast majority of AAA patients are asymptomatic and diagnosis is normally not possible prior to rupture [4].

Until now, most proteomic studies attempting to define AAA biological markers were performed either in AAA tissue samples [5], or in conditioned media containing proteins released from AAA tissue [6], [7], considering that pathological mechanisms take place in a primary aortic blood vessel and that biological changes in aortic tissue will be reflected in those released proteins. Indeed, only one discovery proteomic study has been reported on AAA serum samples [8], which led to the identification of three highly abundant proteins, but none were deemed plausible potential biomarkers of aneurismal disease. Recently, we performed a combined transcriptomic and proteomic study in macrophage extracts that was verified by antibody protein array in the same cell extracts and in plasma of AAA patients [9]. All these proteomic studies for AAA biomarker discovery used profiling techniques such as surface-enhanced laser desorption/ionization time of flight mass spectrometry (MS) or 2-dimensional gel electrophoresis to compare protein abundance between two or more groups of samples.

Our most recent work presented here used a new data-independent (DIA) shotgun proteomics method, PAcIFIC [10], [11] for the quantitative analysis of plasma samples that were depleted of the 14 most abundant proteins. In order to quantify the larger number of proteins, two different quantitative methods were applied: spectral count and TMT isobaric labeling. The quantitative PAcIFIC technology proved to be a powerful tool for the discovery of proteins differentially abundant between pooled plasma samples from AAA and control patients. We present here the results of this study, which is the first high throughput quantitative proteomic study comparing plasma proteomes of patients with and without AAA. This study was performed with the following objectives to: 1) identify and evaluate potential biological markers for AAA screening, which will ensure early diagnosis and subsequent early treatment prior to rupture events which have very high mortality, and 2) provide a better understanding of the pathophysiological mechanisms involved in the evolution of AAA. Our quantitative proteomic strategy allowed the identification of five potential biomarkers of AAA, three of which are proteins involved in the regulation of the kallikrein-kinin system, which others have suggested may play a role in the evolution of AAA.

## Results

**Figure 1:**
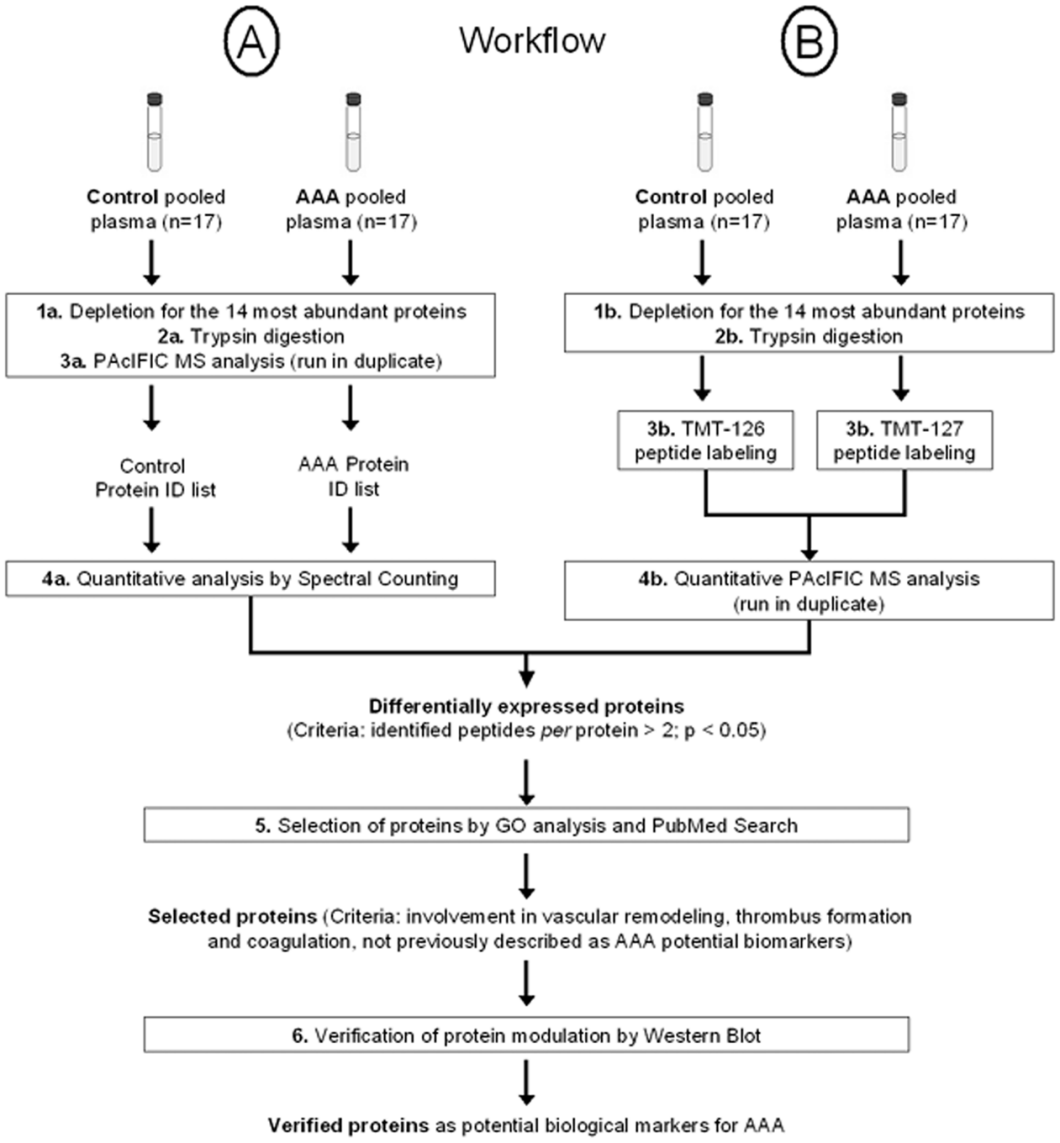
Workflow for the proteomic analysis of protein plasma samples. 17 plasma samples of each group were analyzed by two different quantitative strategies after DIA PAcIFIC MS: (A) spectral count, and (B) tandem mass tag isobaric labeling. After selection, only proteins that were verified by Western Blot were considered as potential biological markers for AAA. ID: identification.

In order to find potential biological markers of AAA, our proteomic strategy (**Fig. 1**) applied a quantitative version of the DIA PAcIFIC method to identify differences in protein abundance between two plasma pools of 17 AAA and 17 control patients each previously depleted of the 14 most abundant proteins.

### Clinical population

There was no statistically significant difference between the baseline characteristics of AAA and control patients selected in this study, except for the presence or absence of AAA (**Table 1**).

**Table 1:** Baseline characteristics of the CORONA population used for the proteomic analysis.

Characteristics	Controls	AAA	Exact p-value
	(n = 17)	(n = 17)	
Age (years), median value [25^th^ to 75^th^ percentile range]	63 [56–71	61 |57–68]	0.75
Gender, n (% male)	17 (100)	17 (100)	ND
BMI (kg/m^2^), median value[25^th^ to 75^th^ percentile range]	26.2 [25–27.8]	27.7 [24.1–29.9]	0.28
**Cardiovascular risk factors, n (%)**			
Type 2 diabetes	5 (29.4)	6 (35.3)	1
Arterial hypertension	9 (52.9)	10 (58.8)	1
Dyslipidemia	10 (58.8)	12 (70.6)	0.72
Smoking (past or current)	13 (76.5	16 (94.1)	0.33
Positive family history for CVD	5 (29.4)	8 (47.1)	0.48
**Personal history of CAD, n (%)**			
Unstable angina pectoris	4 (23.5)	1 (5.9)	0.33
Stable angina pectoris	5 (29.4)	5 (29.4)	ND
Peripheral arterial disease	3 (17.6)	6 (35.3)	0.43
Stroke	1 (5.9)	2 (11.8)	1
Previous myocardial infarction	7 (41.2)	4 (23.5)	0.47
Carotid artery stenosis	5 (29.4)	4 (23.5)	0.69
1- or 2-coronary vessel disease	2 (11.8)	5 (29.4)	0.40
3- or 4-coronary vessel disease	15 (88.2)	12 (70.6)	0.40
**Major medications at time of inclusion, n (%)**			
Aspirin	15 (88.2)	13 (76.47)	0.66
Beta-blockers	13 (76.5)	12 (70.6)	1
ACE inhibitors	4 (23.5)	8 (47.1)	0.28
Calcium antagonists	6 (35.3)	3 (17.6)	0.44
Statins	16 (94.1)	12 (70.6)	0.17
**Presence of other arterial aneurisms, n (%)**			
Iliac aneurismal extension	0(0)	4 (23.5)	0.10
Femoral arterial aneurism	0(0)	1 (5.9)	1
Popliteal arterial aneurism	0(0)	4 (23.5)	0.10

AAA: abdominal aortic aneurysm, ACE: angiotensin converting enzyme, BMI: body mass index, CAD: coronary artery disease, CVD: cardiovascular diseases, ND: no difference between values of both groups of patients.

### MS protein analysis of pooled plasma samples

#### Qualitative analysis from the label-free experiment

Considering only peptide identifications with probability scores ≥0.9 (FDR<1%), duplicate PAcIFIC MS analysis identified approximately one thousand proteins per sample. In the control pooled plasma sample, 328 proteins were identified with at least two peptides (i.e. multiple hit identifications), whereas 667 proteins were identified with only one peptide (single hit identifications). In the AAA pooled plasma sample, 311 proteins were identified as multiple hits while 687 proteins were identified as single hits. Using only the list of proteins identified with multiple unique peptides PAcIFIC analysis provided an apparent dynamic range of detection of 4.5E+06. For example, using representative identified proteins with known plasma concentrations we detected proteins of the following concentrations: 72 kDa type IV collagenase (8.8E+03 pg/mL), L-selectin (1.7E+04 pg/mL), beta-2-microglobulin (1.1E+06 pg/mL), apolipoprotein E (3.4E+07 pg/mL), ceruloplasmin (2.1E+08 pg/mL), alpha-2-macroglobulin (1.4E+09 pg/mL) and albumin (4.0E+10 pg/mL).

#### Biomarker discovery in pooled plasma samples

Quantitative analysis of plasma samples from control and AAA patients performed by PAcIFIC using the label-free spectral counting and the isotopic dilution TMT method are presented in **Supporting information S1**. Only proteins identified by multiple unique peptides were considered. Duplicate normalized spectral counts were used to calculate statistically significant differences in protein ratios (p-value<0.05) between AAA and control samples. Of 308 proteins quantified, 53 proteins appeared to be significantly differentially abundant between AAA and control pooled plasma samples. From these, 18 were up-regulated and 35 down-regulated in AAA pooled plasma samples compared to controls.

The total number of proteins identified in the TMT-duplex experiment was 788, and reporter ion intensity values of 146 proteins could be used to calculate AAA to control protein ratios and 40 proteins appeared to be differentially abundant (p-value<0.05) between AAA and controls. From these, 30 were up-regulated and 10 down-regulated in AAA pooled plasma samples compared to controls.

The two quantitative analyses determined 80 proteins exhibiting a statistically significant change in abundance, of which eight were in common to both methods: complement C1s subcomponent, alpha-2-antiplasmin, and Complement factor H-related protein 1, ceruloplasmin, complement C5, apolipoprotein B-100, inter-alpha-trypsin inhibitor heavy chain H3, inter-alpha-trypsin inhibitor heavy chain H4 . Finally, the last five proteins showed the same change in abundance by both TMT labeling and spectral counting analysis.

### Verification of differentially regulated proteins by Western blot

#### Selection of putative biomarker proteins

The 80 proteins whose change in abundance was found to be statistically significant were classified by GO analysis. From this analysis, 19 proteins were identified in the following three categories as most likely related to putative AAA biological mechanisms: **1)** vascular remodeling (vinculin, alpha-actinin-1, talin-1, gelsolin, adenylyl cyclase-associated protein 1 (CAP1), profilin-1, and filamin-A), **2)** intraluminal thrombus formation and coagulation (protein AMBP, extracellular superoxide dismutase, carboxypeptidase N catalytic chain (CPN1), carboxypeptidase B2, platelet basic protein, alpha-2-antiplasmin, pleckstrin, corticosteroid binding globulin, heparin cofactor 2, and kallistatin), and **3)** inflammatory response (lysozyme C, and adiponectin). Most of these proteins had not previously been directly identified in plasma of AAA patient and so they were followed in individual patient plasma by WB analysis (**Supporting information S1**).

#### Biomarker verification in individual patient plasma

**Figure 2:**
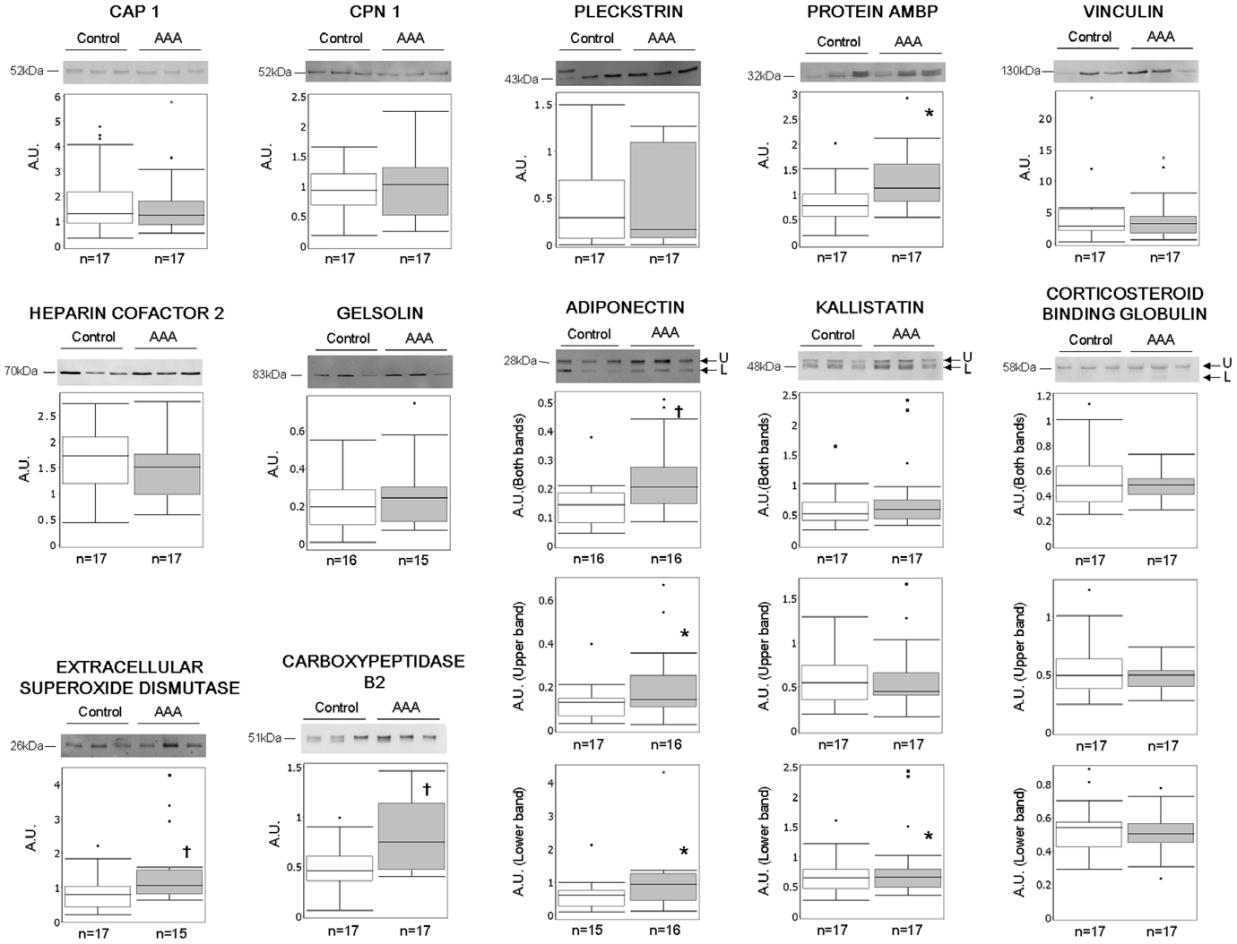
Proteins abundance in individual plasma samples from AAA and control patients. Data of all patients tested is represented in a box plot but only Western blot of three representative samples of each group are presented. The position of Mr value for each protein is indicated. * p<0.05; † p<0.01. n = number of patients in each group; A.U.: arbitrary units; U: upper; L: lower.

Of the 19 proteins chosen, only gelsolin, adiponectin, extracellular superoxide dismutase, corticosteroid-binding globulin, kallistatin, and carboxypeptidase B2 were detected and quantified by WB analysis in crude plasma (**Fig. 2**) of the 34 individual patients' plasma. While gelsolin and corticosteroid-binding globulin did not show any significant difference of protein abundance between AAA and control patients, the remainder did. Specifically, adiponectin, kallistatin, extracellular superoxide dismutase, and carboxypeptidase B2 were detected as being abundant at significantly increased levels in AAA compared to control plasma samples.

CAP1, pleckstrin, vinculin, CPN1, heparin cofactor 2, and protein AMBP were detected and quantified after removal of the 14 most abundant proteins by immunodepletion (**Fig. 2**). Only AMBP appeared to be significantly up-regulated in AAA compared to control plasma samples, as shown by MS quantification. AAA to control ratios of both MS and WB quantification of selected proteins are presented in **Table 2**.

**Table 2:** Comparison between mass spectrometry and western blot analyses.

	Mass Spectrometry		Western Blot		
Protein	SC ratio	TMT ratio	Quantified	Ratio	
	AAA *vs.* Control	AAA *vs.* Control	bands	AAA *vs.* Control	p-value
*Extracellular superoxide dismutase*	*4.87*	*-*	*Unique^1^*	*1.94*	*0.007*
*Protein AMBP*	*1.12*	*-*	*Unique^2^*	*1.90*	*0.037*
Gelsolin	0.81	-	Unique^1^	1.08	0.67
Heparin cofactor 2	0.76	-	Unique^2^	1.02	0.24
Carboxypeptidase N catalytic chain	0.71	-	Unique^2^	1.00	0.63
Alpha-2 antiplasmin	0.63	1.12	ND	NC	NC
Vinculin	0.44	-	Unique^2^	1.45	0.74
Filamin-A	0.40	-	ND	NC	NC
Alpha-actinin-1	0.20	-	ND	NC	NC
Talin-1	0.19	-	ND	NC	NC
Profilin-1	0.16	-	ND	NC	NC
Pleckstrin	0.12 ↓	-	Unique^2^	9.04	0.56
Adenylyl cyclase- associated protein 1	0.01	-	Unique^2^	0.89	0.90
*Adiponectin*	*-*	*1.78*	*Both^1^*	*2.36*	*0.0092*
			*Upper^1^*	*1.87*	*0.033*
			*Lower^1^*	*1.69*	*0.036*
Lysozyme C	-	1.39	ND	NC	NC
*Carboxypeptidase B2*	*-*	*1.35*	*Unique^1^*	*1.45*	*0.0062*
*Kallistatin*	*-*	*0.89*	Both^1^	1.32	0.11
			Upper^1^	1.20	0.47
			*Lower^1^*	*1.31*	*0.046*
Corticosteroid-binding globulin	-	0.72	Both^1^	1.04	0.25
			Upper^1^	1.00	0.23
			Lower^1^	1.10	0.74
Platelet basic protein	-	0.68	ND	NC	NC

SC: spectral count. TMT: tandem mass tag. Proteins verified by western blot analysis are indicated in italic. WB on crude plasma samples^1^. WB on depleted plasma samples^2^. ND: non detected. NC: non calculated.

## Discussion

The aim of the present study was to identify and confirm potential biological markers for AAA screening and to provide a better understanding of the pathophysiological mechanisms involved in the presence of AAA. To accomplish these goals, we used quantitative DIA PAcIFIC analysis to discover proteins differentially abundant between pooled plasma samples from 17 AAA and 17 control patients. GO analysis was performed on the 80 proteins identified as putative biomarkers to further refine the set to be examined by WB analysis on individual patient plasma samples.

The 17 AAA and 17 control patients were selected from 265 recruited in the CORONA study [12]. This sub-population had a well-defined phenotype and the baseline characteristics of AAA and control patients did not present any significant differences except aneurismal pathology, i.e. they seemed to have the same cardiovascular risk factors. Thus, the main interest here was to identify potential biomarkers that discriminate patients with very similar phenotypes. In order to do so WB testing of the potential biomarkers was performed on the same population rather than healthy patients as controls.

To increase detectable proteomic dynamic range, the 14 most abundant proteins were immunodepleted prior to PAcIFIC analysis. The plasma proteome is arguably the most complex human-derived proteome, with a dynamic range of protein concentration of at least 12 orders of magnitude [13], and in normal plasma 22 proteins represent 99% of total protein content [14]. Thus, the challenging analysis of the remaining 1%, known as “deep proteome”, which includes thousands of different proteins [15], involves a mandatory step of depletion of major proteins before sample analysis. A recent study [16] showed that shotgun proteomic analysis of plasma depleted of these most abundant proteins is highly reproducible. Qualitative analysis of pooled plasma samples by PAcIFIC identified proteins (multiple unique peptide hits) over a dynamic range of 4.5E+6 without any previous fractionation of the sample. This outcome is possible because the DIA PAcIFIC analysis circumvents the dynamic range limitations inherent to data-dependent MS based proteomic methods [17]. Furthermore, the DIA PAcIFIC method is compatible with two quantitative methods: spectral counting and TMTs, the latter of which allows samples to be multiplexed [11]. Thus, we have shown, as have others [10], [18], the superiority of DIA methods to extend detectable proteomic dynamic range. In our study, and taking only multiple hit protein identifications, we observed that spectral counting has a dynamic range that is one order of magnitude higher than the TMT isobaric labeling. Hence spectral counting is better for quantification in a broader range of protein concentrations but worse for multiplexing. Quantitative PAcIFIC analysis, using both a label-free spectral count approach and an isotopic labeling multiplexed TMT approach, produced 80 differentially abundant proteins between AAA and control pooled plasma samples. These two approaches were complementary, identifying eight overlapping proteins with significant differences in abundance. This low number of proteins could be due to sample processing for the TMT isobaric tagging method that results in loss of protein or from complexities related to chemical reactions which never yield 100% efficiency and in so doing exacerbate the issue of sample complexity.

GO classification of differentially abundant proteins was performed in order to select a representative protein set for verification by WB analysis. Nineteen proteins out of 80 potentially involved in aneurismal disease were selected for testing in individual patient samples. This technique also made feasible the analysis of individual plasma samples from the 34 patients used for making pools for the discovery proteomic phase of the study. Moreover, WB analysis is an antibody based semi-quantitative method that is similar to the clinical assays that might eventually be used to diagnose AAA from control patients. However, here we note that WB analysis was less sensitive than PAcIFIC analysis as judged by detection of only 12 of 19 selected proteins. Indeed, as others have noted, verification of quantitative MS results during biomarker discovery by existing antibody-based assays represents a serious limitation [19]. This may be accomplished by multiple reaction monitoring [20]–[22], but this is not completely satisfying in that ideally one would like to have an orthogonal method for verification. Despite limitations in sensitivity compared to shotgun proteomics, targeted WB analysis offered supplementary biochemical information of analyzed proteins such as molecular weight and presence of in vivo protein fragments. This was the case for adiponectin and kallistatin, where WB showed two bands that could be explained by the presence of a signal peptide at the N-terminal part of these proteins with molecular masses of 1886 Da and 2203 Da, respectively. These peptides were not observed in MS experiments possibly due to the absence of lysines and arginines until position 33 (for adiponectin) and 50 (for kallistatin) of the entire proteins (**Supporting information S2**)

Of the 12 proteins detected by WB analysis, five appeared to be differentially up-regulated in plasma samples of AAA compared to control patients: adiponectin, extracellular superoxide dismutase, carboxypeptidase B2, kallistatin, and protein AMBP. None of these five proteins have been previously proposed as potential biological markers for AAA diagnosis [23]–[25]. Since only two of the 17 AAA patients used for the present study had infrarenal aortic diameter >50 mm (data not shown), our results may be interpreted as evidence that the five WB verified proteins should be considered as potential biomarkers for early diagnosis of AAA. Of the verified proteins, only kallistatin had a AAA to control ratio by WB analysis that was discordant with the MS result. Indeed, kallistatin appeared to be up-regulated in AAA plasma samples because the trend was observed across all 34 patients by WB whereas the MS result was from a pool, thus highlighting the need for such follow-up experiment to verify MS-based hypotheses. Verification of the other seven proteins detected and quantified probably failed because of inter-individual variability among the human samples. For this subset of proteins, these results could be indeed interpreted as even though the inter-individual variability of humans affects the pooling of samples, our study succeeded in producing near to 42% of true positives.

**Figure 3:**
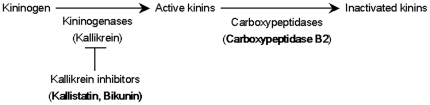
Simplified schematic representation of kallikrein-kinin system regulation. Kininogen is cleaved by kininogenases, such as tissue kallikrein, to generate active kinins. Kallistatin and bikunin are specific inhibitors of tissue kallikrein. Carboxypeptidases, such as carboxypeptidase B2, can cleave C-terminal arginine or lysine residues from active kinins, i.e. bradikinin, to inactivate them thereby regulating their activities.

The five validated proteins were involved in different biological processes (**Supporting information S1**). Adiponectin has cytokine activity and its level in human serum was already described to be increased in early AAA patients (diameter<40 mm) compared to controls [26]. Extracellular superoxide dismutase is involved in superoxide metabolic processes to protect the extracellular space from ROS. It is well known that oxidative stress contributes importantly to the development of AAA [27]–[29]. Carboxypeptidase B2 is an enzyme with metallocarboxypeptidase activity involved in proteolysis. Protein AMBP gives rise, by proteolytic cleavage, to two different proteins (**Supporting information S3**): alpha-1 microglobulin, a lipocalin whose biological functions are not known [30], and bikunin, that, as well as kallistatin, has serine-type endopeptidase inhibitor activity. Moreover, kallistatin was also described as a potent vasodilator [31], consistently with increased concentration of this protein in plasma samples of AAA patients. Importantly, bikunin, i.e. one of the proteolytic products of protein AMBP, together with kallistatin and carboxypeptidase B2 are proteins involved in the regulation of the kallikrein-kinin system (**Fig. 3**). On one hand, the specific inactivation of the proteolytic activity of tissue-kallikrein and plasma-kallikrein can be produced by both kallistatin [32] and bikunin [33]. On the other hand, carboxypeptidase B2 was demonstrated to be responsible for the hydrolysis of bradykinin [34]. Thus, it seems that an increase of kallistatin and bikunin levels may lead to a decrease of active kinins. In the same way, an increase of carboxypeptidase B2 levels may generate an increase on kinin degradation for their subsequent inactivation. Interestingly, it has been demonstrated that kininogen deficiency promotes the development of AAA in kininogen-deficient Brown Norway rats after feeding with a high-fat (atherogenic) diet [35]. Therefore, concerning pathophysiological mechanisms involved in AAA, these results suggest a relationship between decreased levels of active kinins and the presence of AAA.

In conclusion, the present study showed that the combination of plasma depletion of high abundance proteins with DIA PAcIFIC analysis offered a useful tool for the screening of new potential biomarkers of AAA. WB analysis confirmed five PAcIFIC identified proteins as potential biomarkers for early AAA diagnosis (i.e. adiponectin, extracellular superoxide dismutase, kallistatin, carboxypeptidase B2, and protein AMBP). The latter three proteins are known to be involved in the regulation of the kallikrein-kinin system, which was recently suggested to play a role in AAA development. Finally, large cohort studies are needed to validate the application of these five potential biological markers for the screening of AAA in the population at risk. Also, it will be necessary to develop ELISAs for adequate detection and measuring of abundance of these proteins.

## Materials and Methods

### Clinical population and patients matching for the proteomic analysis

CORONA clinical protocol included 265 patients with advanced coronary disease who underwent coronary artery bypass grafting from 2002 to 2006 in University Regional Hospital Center of Lille (France). The ethics committee of the Lille University Hospital Center (France) approved the study, and each patient provided written informed consent (CP 01/96 of 04/12/01). Vascular and abdominal echography examination was performed after surgery to check the quality of bypass and the presence of AAA by measuring the aorta diameter. At the time of recruitment, AAA was detected in 17 patients (6.4% of the population), considering aortic diameter ≥30 mm or infra- to supra-renal ratio >1.5. Venous blood samples were obtained from all patients the day before the surgery.

The 17 AAA patients were paired with 17 controls, according to their age, gender, tobacco consumption, arterial hypertension, diabetes and dyslipidemia. Age distributions of plasma samples from selected control and AAA patients were similar over the time (data not shown). The biological and clinical data of these 34 patients were analyzed by exact nonparametric inference tests (StatXact.8 software) and permutation test was used for continuous data. For categorical data, Fisher's exact test and Pearson's Chi-square test were used to analyze binomial and multinomial data, respectively.

### Plasma sample treatment

Plasma samples (EDTA as anticoagulant) were collected within 2 h from centrifugation (1109 g, 15 min at room temperature) of 7.5 mL blood sample. Plasma samples were divided into 300 µL aliquots and stored at −80°C. They did not undergo more than two freeze/thaw cycles before processing for proteomic analysis.

A pool of both groups of patients (17 AAA and 17 controls) was prepared using 50 µL of plasma per patient. Two-hundred µl of each pool and 70 µL of individual plasma samples (for WB) were depleted for the 14 most abundant proteins using Multiple Affinity Removal System Hu-14 (MARS-14) spin cartridge (Agilent Technologies, Santa Clara, CA) according to the manufacturer's protocol. After depletion, pooled and individual plasma samples were concentrated in 500 µL of 100 mM ammonium bicarbonate using spin concentrators (Agilent Technologies, Santa Clara, CA), and protein concentration was determined by Bradford assay (Biorad, Hercules, CA).

Urea (6 M final concentration) was added to depleted plasma pools before reduction with 2.5 µL of 200 mM dithiothreitol at 37°C for 1 h, and alkylation with 11.25 µL of 200 mM iodoacetamide at 37°C for 1 h in the dark. Samples were then diluted three times with 100 mM ammonium bicarbonate before addition of trypsin (enzyme to protein ratio of 1∶50). Digestion was carried out overnight at 37°C. Peptides of digests were desalted on C18 macrospin columns (The Nest Group, Southborough, MA) according to the manufacturer's instructions and stored at −80°C until PAcIFIC MS analysis was performed.

### Peptide labeling and PAcIFIC MS

Each pooled plasma sample was run in duplicate for both PAcIFIC and quantitative PAcIFIC MS analyses. For PAcIFIC MS, peptide samples were dried with a vacuum concentrator and resuspended in 5% acetonitrile, 0.1% formic acid to reach an estimated peptide concentration of 0.5 µg/µL. HPLC was performed on a Waters NanoAcquity (Manchester, U.K.) system using homemade columns and precolumns as previously described [36]. An estimated amount of 1 µg of plasma peptides was used for each sample injection. A LTQ XL linear ion trap mass spectrometer (ThermoFisher, San Jose, CA) was used to perform PAcIFIC MS analysis as previously described [10]. Briefly, in a first injection, PAcIFIC acquisition used 15 consecutive collision-induced dissociation (CID) MS/MS scans of m/z = 401.5, 403, 404.5, 406, 407.5, 409, 410.5, 412, 413.5, 415, 416.5, 418, 419.5, 421 and 422.5 with an isolation width set to 2.5 and collision energy to 35%. In the consecutive range, m/z values from 422.5 to 445 were analyzed, and so on until m/z = 1412.5 was reached (45 repeated injections in total).

Tandem mass tag (TMT) duplex approach [37] was used for quantitative PAcIFIC MS [11]. Peptide mixtures corresponding to control and AAA pooled plasma were labeled with TMT2-126 and TMT2-127 (ThermoScientific, Rockford, IL) respectively, according to manufacturer's protocol. Labeled samples were combined, dried in a vacuum concentrator and dissolved in 5% acetonitrile, 0.1% formic acid to reach an estimated peptide concentration of 0.5 µg/µL. MS and HPLC conditions were the same as described above. Quantitative PAcIFIC MS analysis was applied as described above with the following modifications: between each CID fragmentation scan, a zoomed pulsed-Q dissociation scan with collision energy set to 35%, activation Q = 0.55, and activation time = 0.4 was performed from m/z = 121 to 132 in order to measure the TMT reporter ions.

### Data processing and quantification of PAcIFIC MS

For each PAcIFIC or quantitative PAcIFIC replicate, RAW files were converted to mzXML files (45 files in total) and peak lists were generated and searched with Sequest against the IPI human database v3.49 (74017 entries; http://www.ebi.ac.uk/IPI/IPIhuman.html). The following settings were used: proteolytic enzyme: trypsin; number of missed cleavages permitted: 1; precursor tolerance: 3.75 Da; fixed modification: alkylated cysteines; and variable modification: oxidized methionine. Sequest results were converted to pepXML files and probability assessments of identified peptides were computed with PeptideProphet (ISB, Seattle, WA). For all individual searches (45 pepXML files or 1 replicate), peptides with probability scores equal or higher than 0.9 were used (estimated false discovery rate (FDR)<1%). PAcIFIC dynamic range for protein identification was calculated only considering proteins identified as multiple hits (with at least 2 unique peptides), with FDR<1%, and using known plasma protein concentration values, described by the HUPO Plasma Proteome Project [38]. For searches on TMT-labeled samples, the TMT modification was added as fixed, and the intensities of TMT reporter ions (*m/z* = 126 and *m/z* = 127) were extracted from raw data files. Intensity ratios were calculated and normalized to 126-TMT reporter ion using the program Libra (ISB, Seattle, WA). For each protein, the ratio was calculated as a mean value of all intensity ratios of peptides identified. For all searches, when peptides matched to multiple members of a protein family, identification is represented by only one name of proteins or isoforms included in the identified family.

Protein quantifications were calculated only in multiple hit proteins (with at least 2 unique peptides) and with FDR<1%. For spectral count analysis [39], number of spectra *per* protein were computed with ProteinProphet (ISB, Seattle, WA). Datasets of MS/MS data with significant identification assignments were exported into Excel and average spectral counts of duplicates were calculated and normalized to the sum of all spectra from the sample with the highest number of spectra, i.e. AAA plasma sample. In order to avoid missing values when a protein was not identified in one group (control or AAA), an arbitrary value of 0.1 was summed to the number of spectral counts. Then, G-test was applied to spectral counts of each protein in every sample in order to evaluate differences of protein abundance [40]. P-values<0.05 were considered as significant.

Datasets of MS/MS data containing mean values of intensity ratios of TMT reporter ions were exported into Excel. Then, t-test was applied to duplicated values of intensity ratios for each protein in order to evaluate differences of protein abundance. Significant proteins were considered when p-value<0.05 and fold change >12%, since variation in technical replicates for isobaric labeling has been estimated to that value [41], [42].

### Selection of proteins to be verified

Gene Ontology (GO) annotations of significant differentially abundant proteins were automatically assigned with the freely available internet tool Protein Information and Property Explorer [43]. Assignments were manually filtered according to the biological implications in the pathology, and validated using the GO Consortium tool AmiGO Version 1.7 and GO database release of 2010-07-31 [44]. According to this classification, proteins to be verified were selected in order to cover the wide variety of biological processes and molecular functions that the literature indicates may be involved in AAA biological mechanisms such as 1) vascular remodeling, 2) intraluminal thrombus formation and coagulation, and 3) inflammatory response. Lastly, we focused on proteins which had not been previously identified in plasma of AAA patients.

### Western blot analysis

WB analysis was performed on the same 17 AAA and 17 control individual plasma samples that were used for the proteomic analysis. Plasma proteins (depleted or not with human MARS-14) were diluted with 38 µL of RIPA buffer and separated by SDS-PAGE (12% acrylamide gels for all proteins except for adiponectin (15%) and vinculin (6%)). Then, proteins were electrotransferred onto a 0.45 µm Hybond nitrocellulose membrane (GE Healthcare), except for corticosteroid-binging globulin, AMBP and pleckstrin, for which a PVDF membrane (Sigma Aldrich) was used. Visual verification of total protein loads was performed by Ponceau staining of the membranes. Primary antibodies, that were diluted in 5% w/v non fat dry milk in TBS-Tween and used at 4°C, overnight, were: polyclonal rabbit anti human adiponectin (2 µL crude plasma, 1∶1000 v/v, Abcam); monoclonal mouse anti human gelsolin (2 µL crude plasma, 1∶5000 v/v, Sigma Aldrich); polyclonal rabbit anti human corticosteroid-binding globulin (2 µL crude plasma, 1∶500 v/v, Proteintech group inc); polyclonal rabbit anti human extracellular superoxide dismutase [Cu-Zn] (2 µL crude plasma, 1∶100 v/v, NovusBiological); polyclonal goat anti human kallistatin (2 µL crude plasma, 1∶2000 v/v, R&D systems); monoclonal mouse anti human carboxypeptidase B2 (2 µL crude plasma, 1∶1000 v/v, Abcam); polyclonal goat anti human pleckstrin (25 µg depleted plasma, 1∶1000 v/v, Abcam); polyclonal rabbit anti human CPN1 (25 µg depleted plasma, 1∶500 v/v, Abcam); polyclonal rabbit anti human CAP1 (25 µg depleted plasma, 1∶500 v/v, Abcam); monoclonal mouse anti human AMBP (10 µg depleted plasma, 1∶500 v/v, Abnova); monoclonal mouse anti human vinculin (10 µg depleted plasma, 1∶100 v/v, Abcam); and monoclonal mouse anti human serpin D1 (5 µg depleted plasma, 1∶2000 v/v, Abcam).

Secondary antibodies, that were diluted 1∶5000 v/v in 5% w/v non fat dry milk in TBS-Tween and used at room temperature for 1.5 h, were: donkey anti goat (Abcam), ECL mouse IgG-HRP (GE Healthcare), and ECL rabbit IgG-HRP (GE Healthcare). Finally, the specific proteins were detected using ECL Plus Western blotting detection reagent (GE Healthcare) followed by membrane scanning with an Ettan DIGE Imager scanner (GE Healthcare) at excitation/emission wavelengths of 480 nm/530 nm to yield images with a pixel size of 100 µm.

Quantity One software (Biorad) was used for the acquisition of intensity values of detected proteins from blot images. Normalized (inter and intra membrane) intensity values were used to evaluate differences between protein abundance levels in AAA compared to control samples applying permutation test for nonparametric inference (StatXact.8 software). Median values of normalized AAA and control intensities were used to calculate AAA to control ratio per membrane. For each protein, overall AAA to control ratio was calculated as the mean value of AAA to control ratios per membrane.

## Supporting Information

Supporting Information S1 **Quantitative MS analysis of plasma proteins from AAA and control patients.**
(DOC)

Supporting Information S2 **Detailed aminoacid sequences of adiponectin and kallistatin.** Sequences identified by MS are indicated in blue. (**A**) There is not “K” or “R” in the signal peptide sequence of adiponectin to allow identification by MS. The first “K” or “R” in the sequence appears at position 33. (**B**) There is not “K” or “R” in the signal peptide sequence of kallistatin to allow identification by MS. The first “K” or “R” in the sequence appears at position 50.
(TIF)

Supporting Information S3 **Detailed aminoacid sequence of the proteolytic products of AMBP protein.** Alpha-1-microglobulin has a sequence of 183 aa, and MS identification by PAcIFIC covered 57.9% of the sequence (aminoacid indicated in blue) with 30.8% of the total spectral counts corresponding to AMBP protein. Bikunin has a sequence of 147 aa, and MS identifications by PAcIFIC covered 61.2% of the sequence (aminoacid indicated in blue) with 69.2% of the total spectral counts corresponding to AMBP protein. 22 23 24 25 26 27 28 29 30 31 32 33 34 35.
(TIF)
