# Supplementary material for: Quantitative Mass Spectrometry Analysis Using PAcIFIC for the Identification of Plasma Diagnostic Biomarkers for Abdominal Aortic Aneurysm
Source: PLoS One. 2011 Dec 7;6(12):e28698. doi: 10.1371/journal.pone.0028698 (PMC3233585; doi:10.1371/journal.pone.0028698)
Supplement: Supporting Information S1 — Quantitative MS analysis of plasma proteins from AAA and control patients. (DOC) [file pone.0028698.s001.doc]

### .**Supplementary information S1. Quantitative MS analysis of plasma proteins from AAA and control patients.**

| **Accession number*** | | Protein name | **Spectral count** | |  | **TMT isobaric labeling** | |
| --- | --- | --- | --- | --- | --- | --- | --- |
|  | |  | **p** | **Ratio** |  | **p** | **Ratio** |
| **GO:0007155 Cell adhesion** | | | | | | | |
| *P02760* | *Protein AMBP* | | *0.037* | *1.12* |  | *-* | *-* |
| *P18206* | *Vinculin* | | *0.026* | *0.44* |  | *-* | *-* |
| Q9P0K1 | Disintegrin and metalloproteinase domain-containing protein 22 | | 0.040 | 0.03 |  | - | - |
| P04004 | Vitronectin | | - | - |  | 0.00038 | 1.26 |
| P35858 | Insulin-like growth factor-binding protein complex acid labile subunit | | - | - |  | 0.044 | 0.89 |
| **GO:0006915 Apoptosis** | | | | | | | |
| *P12814* | *Alpha-actinin-1* | | *0.015* | *0.20* |  | *-* | *-* |
| P08571 | Monocyte differentiation antigen CD14 | | - | - |  | 4.32E-06 | 1.24 |
| **GO:0006801 Superoxide metabolic process** | | | | | | | |
| *P08294* | *Extracellular superoxide dismutase [Cu-Zn]* | | *0.035* | *4.87* |  | *-* | *-* |
| **GO:0006730 One-carbon compound metabolic process** | | | | | | | |
| P00915 | Carbonic anhydrase 1 | | 0.00042 | 0.13 |  | - | - |
| **GO:0030212 Hyaluronan metabolic process** | | | | | | | |
| P19827 | Inter-alpha-trypsin inhibitor heavy chain H1 | | 2.18E-06 | 0.84 |  | - | - |
| P19823 | Inter-alpha-trypsin inhibitor heavy chain H2 | | 5.36E-11 | 1.34 |  | - | - |
| Q06033 | Inter-alpha-trypsin inhibitor heavy chain H3 | | 0.00026 | 1.41 |  | 0.0047 | 1.29 |
| **GO:0006629 Lipid metabolic process** | | | | | | | |
| P04114 | Apolipoprotein B-100 | | 0.00031 | 1.14 |  | 0.021 | 1.24 |
| P02654 | Apolipoprotein C-I | | - | - |  | 0.0082 | 1.22 |
| P80108 | Phosphatidylinositol-glycan-specific phospholipase D | | - | - |  | 0.042 | 1.33 |
| **GO:0006508 Proteolysis** | | | | | | | |
| *P15169* | *Carboxypeptidase N catalytic chain* | | *0.018* | *0.71* |  | *-* | *-* |
| Q76LX8 | A disintegrin and metalloproteinase with thrombospondin motifs 13 | | 0.017 | 0.23 |  | - | - |
| P09871 | Complement C1s subcomponent | | 0.010 | 0.86 |  | 0.012 | 1.14 |
| P00751 | Complement factor B | | - | - |  | 0.011 | 1.19 |
| *Q96IY4* | *Carboxypeptidase B2* | | *-* | *-* |  | *0.00021* | *1.35* |
| P06681 | Complement C2 | | - | - |  | 0.021 | 1.27 |
| P00746 | Complement factor D | | - | - |  | 0.035 | 1.68 |
| **GO:0050873 Brown fat cell differentiation** | | | | | | | |
| P02750 | Leucine-rich alpha-2-glycoprotein | | 0.035 | 1.37 |  | - | - |
| **GO:0045650 Negative regulation of macrophage differentiation** | | | | | | | |
| *Q15848* | *Adiponectin* | | *-* | *-* |  | *0.0059* | *1.78* |
| P02747 | Complement C1q subcomponent subunit C | | - | - |  | 0.0049 | 1.36 |
| **GO:0008283 Cell proliferation** | | | | | | | |
| P01344 | Insulin-like growth factor II | | - | - |  | 0.042 | 0.84 |
| **GO:0007010 Cytoskeletal organization** | | | | | | | |
| *Q9Y490* | *Talin-1* | | *7.47E-08* | *0.19* |  | *-* | *-* |
| P35527 | Keratin, type I cytoskeletal 9 | | 1.87E-04 | 0.18 |  | - | - |
| *P06396* | *Gelsolin* | | *2.49E-04* | *0.81* |  | *-* | *-* |
| *Q01518* | *Adenylyl cyclase-associated protein 1* | | *0.00093* | *0.01* |  | *-* | *-* |
| *P07737* | *Profilin-1* | | *0.035* | *0.16* |  | *-* | *-* |
| *P21333* | *Filamin-A* | | *0.00030* | *0.40* |  | *-* | *-* |
| **GO:0031667 Response to nutrient levels** | | | | | | | |
| P05543 | Thyroxine-binding globulin | | - | - |  | 0.0034 | 1.17 |
| **GO:0006935 Chemotaxis** | | | | | | | |
| *P02775* | *Platelet basic protein* | | *-* | *-* |  | *0.0021* | *0.68* |
| P01031 | Complement C5 | | 0.045 | 1.12 |  | 0.042 | 1.17 |
| **GO:0006979 Response to oxidative stress** | | | | | | | |
| P49908 | Selenoprotein P | | - | - |  | 0.043 | 0.86 |
| **GO:0006954 Inflammatory response** | | | | | | | |
| *P61626* | *Lysozyme C* | | *-* | *-* |  | *0.02* | *1.39* |
| **GO:0006953 Acute-phase response** | | | | | | | |
| P01011 | Alpha-1-antichymotrypsin | | 1.87E-06 | 0.87 |  | - | - |
| P02765 | Alpha-2-HS-glycoprotein | | 0.00013 | 0.87 |  | - | - |
| *P08697* | *Alpha-2-antiplasmin* | | *8.77E-08* | *0.63* |  | *0.0069* | *1.12* |
| Q14624 | Inter-alpha-trypsin inhibitor heavy chain H4 | | 0.013 | 1.18 |  | 0.032 | 1.22 |
| P35542 | Serum amyloid A-4 protein | | - | - |  | 0.038 | 0.85 |
| **GO:0007586 Digestion** | | | | | | | |
| P19961 | Alpha-amylase 2B | | 0.013 | 0.02 |  | - | - |
| **GO:0044419 Interspecies interaction between organisms** | | | | | | | |
| P23142 | Fibulin-1 | | 0.042 | 1.88 |  | - | - |
| **GO:0006094 Gluconeogenesis** | | | | | | | |
| P04406 | Glyceraldehyde-3-phosphate dehydrogenase | | 0.0086 | 0.02 |  | - | - |
| **GO:0007517 Muscle organ development** | | | | | | | |
| P60660 | Myosin light polypeptide 6 | | 0.019 | 0.02 |  | - | - |
| P37802 | Transgelin-2 | | 0.012 | 0.24 |  | - | - |
| **GO:0007242 Intracellular signaling cascade** | | | | | | | |
| *P08567* | *Pleckstrin* | | *0.0070* | *0.12* |  | *-* | *-* |
| **GO:0007601 Visual perception** | | | | | | | |
| Q12805 | EGF-containing fibulin-like extracellular matrix protein 1 | | - | - |  | 0.00066 | 1.2 |
| **GO:0006810 Transport** | | | | | | | |
| *P08185* | *Corticosteroid-binding globulin* | | *-* | *-* |  | *3.20E-06* | *0.72* |
| **GO:0060191 Regulation of lipase activity** | | | | | | | |
| P02655 | Apolipoprotein C-II | | 0.027 | 1.37 |  | - | - |
| P02656 | Apolipoprotein C-III | | - | - |  | 0.043 | 1.4 |
| **GO:0030003 Cellular cation homeostasis** | | | | | | | |
| P00450 | Ceruloplasmin | | 0.012 | 1.07↑ |  | 0.0088 | 1.27 |
| P02790 | Hemopexin | | - | - |  | 0.015 | 1.16 |
| **GO:0050880 Regulation of blood vessel size** | | | | | | | |
| P68871 | Hemoglobin subunit beta | | - | - |  | 0.032 | 0.76 |
| **GO:0007596 Blood coagulation** | | | | | | | |
| P13987 | CD59 glycoprotein | | 0.0027 | 9.70 |  | - | - |
| *P05546* | *Heparin cofactor 2* | | *0.0011* | *0.74* |  | *-* | *-* |
| P01033 | Metalloproteinase inhibitor 1 | | 0.040 | 0.03 |  |  |  |
| P02748 | Complement component C9 | | - | - |  | 0.00059 | 1.21 |
| P01008 | Antithrombin III | | - | - |  | 0.023 | 1.13 |
| P07225 | Vitamin K-dependent protein S | | - | - |  | 0.032 | 0.89 |
| P22891 | Vitamin K-dependent protein Z | | - | - |  | 0.04 | 0.47 |
| **GO:0006956 Complement activation** | | | | | | | |
| P13671 | Complement component C6 | | 0.042 | 1.19 |  | - | - |
| P06310 | Ig kappa chain V-II region RPMI 6410 | | 0.0041 | 0.02 |  | - | - |
| Q03591 | Complement factor H-related protein 1 | | 0.00021 | 0.01 |  | 0.0075 | 1.23 |
| Q9BXR6 | Complement factor H-related protein 5 | | - | - |  | 0.016 | 1.28 |
| P04003 | C4b-binding protein alpha chain | | - | - |  | 0.038 | 1.18 |
| P02745 | Complement C1q subcomponent subunit A | | - | - |  | 0.017 | 1.15 |
| **GO:0030195 Negative regulation of blood coagulation** | | | | | | | |
| P01042 | Kininogen-1 | | 8.75E-07 | 0.74 |  | - | - |
| P04264 | Keratin, type II cytoskeletal 1 | | 1.45E-05 | 0.27 |  | - | - |
| **Non classified** | | | | | | | |
| P04217 | Alpha-1B-glycoprotein | | 0.00062 | 1.16 |  | - | - |
| P07358 | Complement component C8 beta chain | | 0.0015 | 0.73 |  | - | - |
| P08519 | Apolipoprotein(a) | | 7.90E-11 | 0.20 |  | - | - |
| P01876 | Ig alpha-1 chain C region | | 1.65E-05 | 0.18 |  | - | - |
| P01880 | Ig delta chain C region | | 0.0059 | 0.02 |  | - | - |
| Q8N355* | IGL@ protein | | 6.78E-07 | 0.01 |  | - | - |
| P36980 | Complement factor H-related protein 2 | | 1.36E-10 | 0.003 |  | - | - |
| B4DPQ0* | cDNA FLJ54471, highly similar to Complement C1r subcomponent | | - | - |  | 0.044 | 1.12 |
| *P29622* | *Kallistatin* | | *-* | *-* |  | *0.044* | *0.89* |
| B4E3S6* | cDNA FLJ58413, highly similar to Complement component C7 | | - | - |  | 0.047 | 0.76 |

Assignment of UniProt accession numbers were made according to UniProt Knowledgebase Release 15.8 of Sep 22, 2009. Ratio: AAA/control. *Accession number refers to UniProtKB/TrEMBL database instead of UniProtKB/Swiss-Prot. Proteins selected for verification by Western blot are indicated in italics.
