# Supplementary figures and images for: Quantitative Mass Spectrometry Analysis Using PAcIFIC for the Identification of Plasma Diagnostic Biomarkers for Abdominal Aortic Aneurysm
Source: PLoS One. 2011 Dec 7;6(12):e28698. doi: 10.1371/journal.pone.0028698 (PMC3233585; doi:10.1371/journal.pone.0028698)

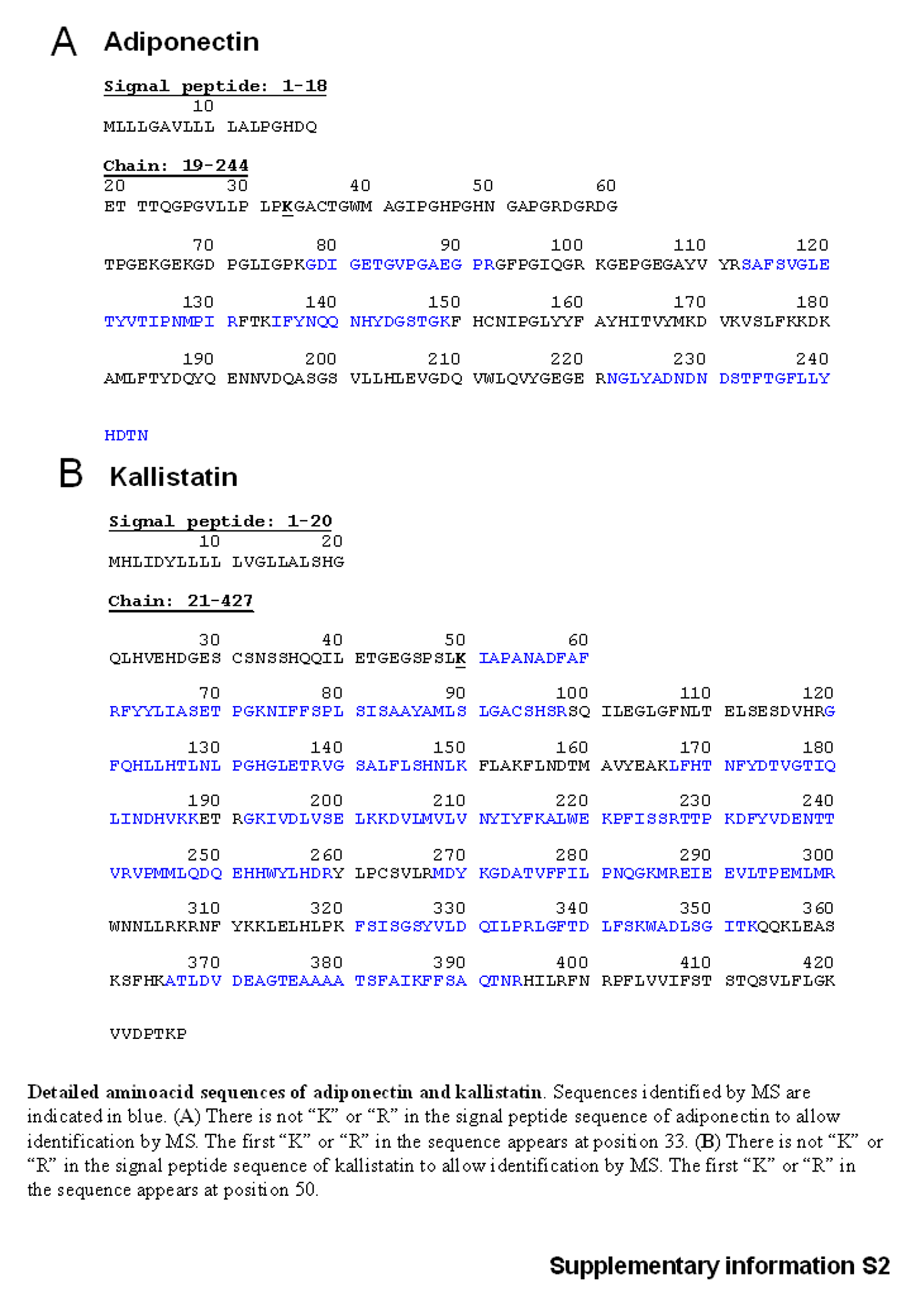

Supplement: Supporting Information S2 — Detailed aminoacid sequences of adiponectin and kallistatin. Sequences identified by MS are indicated in blue. (A) There is not “K” or “R” in the signal peptide sequence of adiponectin to allow identification by MS. The first “K” or “R” in the sequence appears at position 33. (B) There is not “K” or “R” in the signal peptide sequence of kallistatin to allow identification by MS. The first “K” or “R” in the sequence appears at position 50. (TIF) [file pone.0028698.s002.tif]

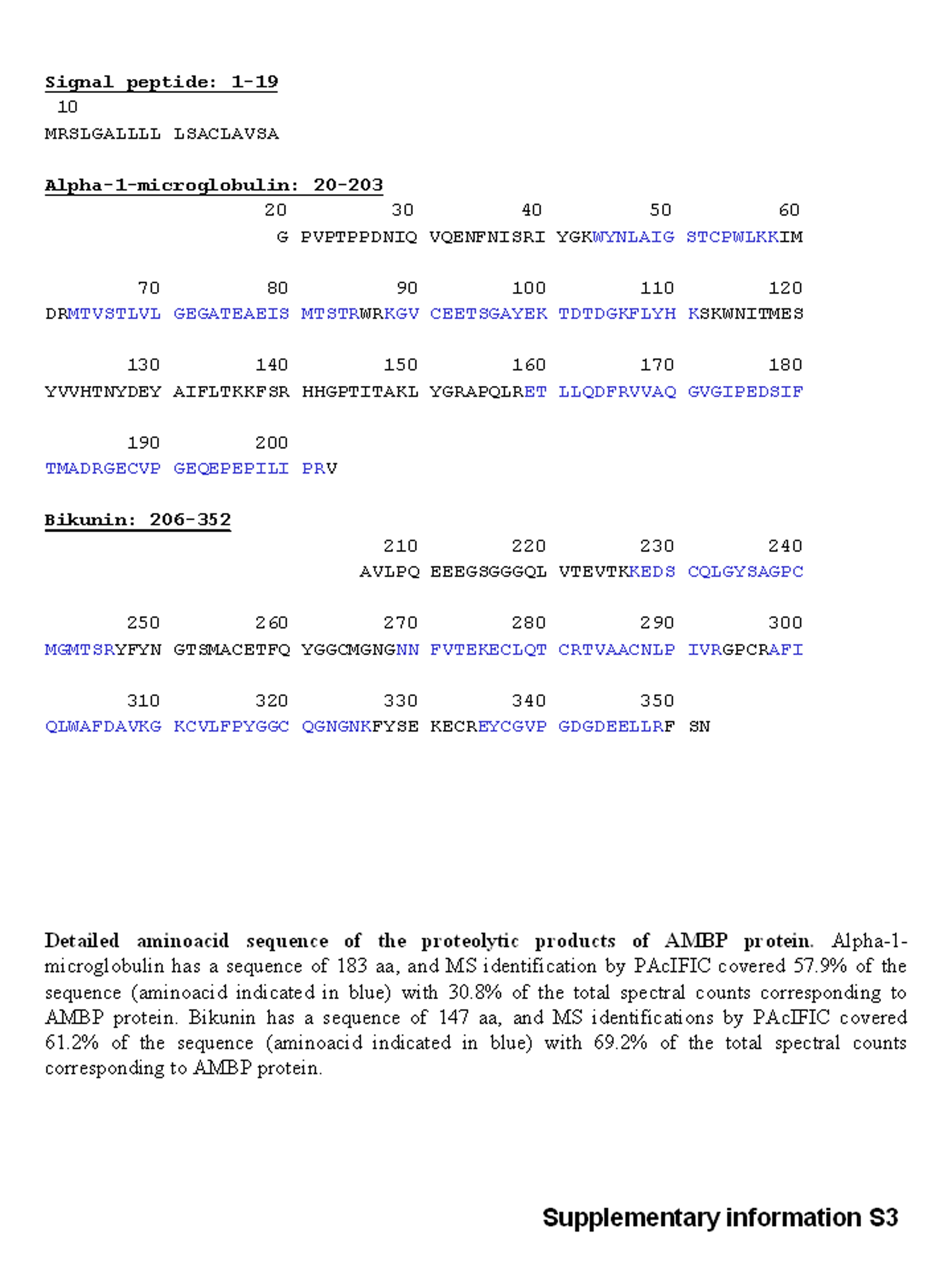

Supplement: Supporting Information S3 — Detailed aminoacid sequence of the proteolytic products of AMBP protein. Alpha-1-microglobulin has a sequence of 183 aa, and MS identification by PAcIFIC covered 57.9% of the sequence (aminoacid indicated in blue) with 30.8% of the total spectral counts corresponding to AMBP protein. Bikunin has a sequence of 147 aa, and MS identifications by PAcIFIC covered 61.2% of the sequence (aminoacid indicated in blue) with 69.2% of the total spectral counts corresponding to AMBP protein. 22 23 24 25 26 27 28 29 30 31 32 33 34 35. (TIF) [file pone.0028698.s003.tif]
